# Exploring Empathy: A Report on How Visual Art Can Foster Understanding and Compassion

**DOI:** 10.7759/cureus.90175

**Published:** 2025-08-15

**Authors:** Anzhelika Ivasenko

**Affiliations:** 1 Physiology, Alfaisal University, College of Medicine, Riyadh, SAU

**Keywords:** cognitive empathy, medical physiology, optometry students, pre-clinical training, visual art

## Abstract

Empathic communication and perspective-taking are essential skills for any healthcare provider, and they must be developed during the training process. Numerous studies have demonstrated the effectiveness of incorporating the humanities into teaching soft skills. We designed an art project in the course of Medical Physiology for optometry students to help them understand how patients with different eye diseases and chronic somatic conditions perceive the world. This is a post hoc quasi-experimental study. The assignment "See the World Through My Eyes" was part of the Medical Physiology program. A total of 156 first-year optometry students were enrolled in the program from 2021 to 2023 and completed the assignment. The students were supposed to choose an art object from a copyright-free museum's online collection and transform it to reflect how patients might perceive it. Post-project feedback was used to evaluate the effectiveness of this intervention. Approximately 65% of participants reported a significant knowledge increase, and 77% reported a substantial enhancement in perspective-taking. The common topics emerging from the narrative reflections were "clinical case presentation," "patient's perspective: understanding and explaining to others," "assumptions/stigmatization," "preparation for future profession," "pathological mechanisms," and "teamwork/collaborations." We believe that the project will help enhance observation and interpretation skills, create awareness of patients' possible visual abnormalities, promote empathy, compassion, and perspective-taking, and allow students to recognize assumptions and mitigate biases.

## Introduction

Empathy was defined by Lemogne et al. [1] as the ability to share one's emotions without confusion between self and others. Clinical empathy is closely linked to patients' compliance with treatment and therapeutic outcomes. Along with affective ("showing empathy") and cognitive ("putting oneself in the patient's shoes"), it also has motivational and behavioral components, i.e., determination and ability to act on understanding the patient's situation, circumstances, and feelings [2]. Clinical empathy is considered a prerequisite for medical professionalism [2], as empathic communication determines the formation of a therapeutic bond or alliance between the patient and the physician and positively influences treatment outcomes, patient satisfaction with care, and physicians' burnout rates.

Systematic reviews by Fragkos and Crampton [2] and Zhou et al. [3] showed the variety of methods and modalities used to teach empathy: experiential training, didactic methods, skills training, role-playing, mixed methods, communication skills training with behavior-based workshops, group discussions on personal experiences and/or simulated scenarios including role-play and simulated patients, role-play, the use of the arts and humanities including poetry and literature, drawings and paintings, reflective writing, cultural studies and history, film, photography, and comics.

Visual and other forms of art have been incorporated into the health professions curriculum over the past two decades, as they have proven effective in teaching empathy, observational skills, and empathetic communication [2-6].

The objective of this pilot project was to explore the potential of visual art in promoting the cognitive component of empathy development in first-year optometry students at KYCO (Kentucky College of Optometry). To achieve this objective, the assignment "See the World Through My Eyes" was incorporated into the Medical Physiology course.

## Technical report

This is a post hoc quasi-experimental study. The assignment "See the World Through My Eyes" was part of the Medical Physiology program. A total of 156 first-year optometry students were enrolled in the program from 2021 to 2023 and completed the assignment. The University of Pikeville IRB approved the research as low-risk (approval number: PROTOCOL_24_0012).

Students were randomly assigned to groups of three to four using the Canvas Learning Management System's random assignment feature. Group leaders were selected randomly within each group. Each group was assigned a clinical condition (ocular diseases, neurologic, endocrine, cardiovascular, or metabolic disorders) for which they had to create a case and explain the mechanisms of vision impairment in their patient. Students were advised to choose an art object from the online collections of copyright-free museums [7] and transform it to reflect how patients might perceive it. They had to create a patient's story, including personal information, and show how patients' daily lives and work are affected by their visual impairment. They had to present the story from the first person (patient).

The post hoc assessment of project effectiveness in reaching the objectives (understanding the mechanisms of vision impairment and perspective-taking) and the level of students' satisfaction with the project was conducted on Canvas in the form of an anonymous survey using four questions on a five-point Likert scale and reflective narrations.

Below are some of the patient cases created by the students, along with the artwork modified to reflect vision impairment in each case. The personal information of all patients, such as names, ages, occupations, and other personal data, was generated by the students specifically for this project and is not associated with real people. Any coincidences with real individuals are unintentional.

Case 1. Protanopia

Patient: Kyle Williams, a 20-year-old student.

Kyle was diagnosed with protanopia at the age of four after he failed to correctly identify the numbers on the Ishihara plates during a color vision screening at school.

This is what Kyle says about his condition: "When I was young, I faced some difficulty distinguishing colors, especially red and green. With age, I learned to recognize certain shades from others. I find that my vision impairment has always affected my perception of the foods I eat, as it can be tricky to pick a ripe banana or tomato and to cook meats thoroughly. When I drive to and from work, I've learned to memorize signs and stoplight patterns. I've been able to purchase apps for my phone, tablet, and TV that use a special filter to minimize defects and I am considering purchasing special glasses to help daily. I do not have concerns about my color blindness limiting my career options in graphic design; it gives a nice unique touch to my work. I do have others look at my work; my professors have been very supportive and helpful in this aspect. My professor even helped me get a photography internship, so it has not caused any issues so far!"

**Figure 1:**
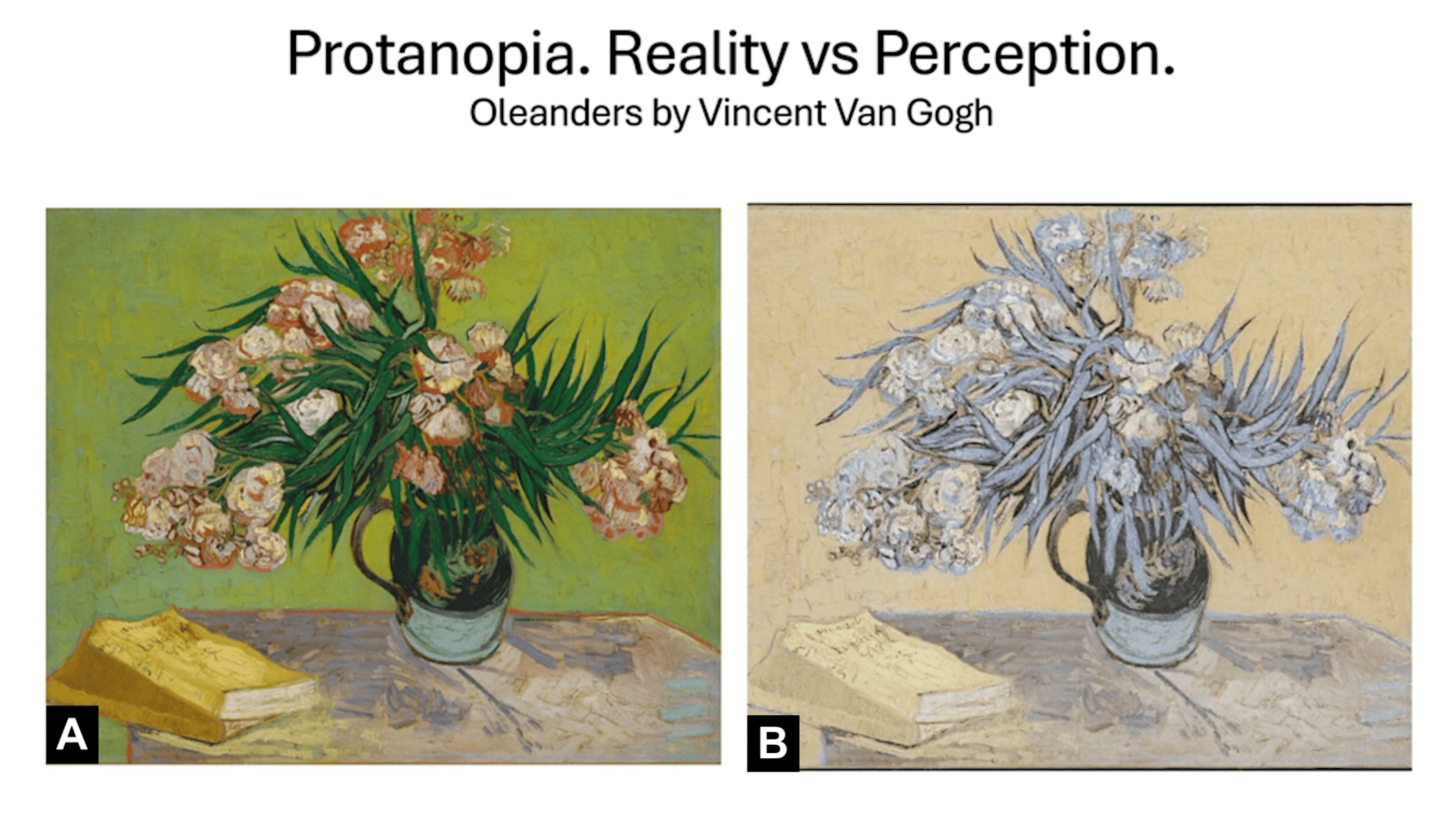
Reality vs. perception for a patient with protanopia. The image was generated by the students as part of the project. (A) "Oleanders" by Vincent Van Gogh [8], courtesy of The Metropolitan Museum of Art, New York, released under Creative Commons Zero (CC0). (B) The same image modified by the students with the help of Open Art AI to reflect the perception of a patient with protanopia.

The type of visual impairment experienced by this patient is presented in Figure 1.

Case 2. Left abducens nerve paralysis

Patient: Richard Johnson, a 60-year-old photographer.

Richard has had poorly controlled hypertension and high cholesterol for five years. Three months ago, he had a stroke and developed left abducens nerve paralysis. This is what Richard says about his condition: "I have always looked through the camera with my right eye, so it has not affected my career as a photographer. I have learned the hard way to always walk with just the right eye open or I will lose balance and lean towards my left side. I decided the only way to give others a glimpse into my life was to take photographs. I learned how to edit my photos to make it seem like the camera was seeing double…. I felt like it was important to educate others because everyone deserves to be able to go out in public without getting stares."

**Figure 2:**
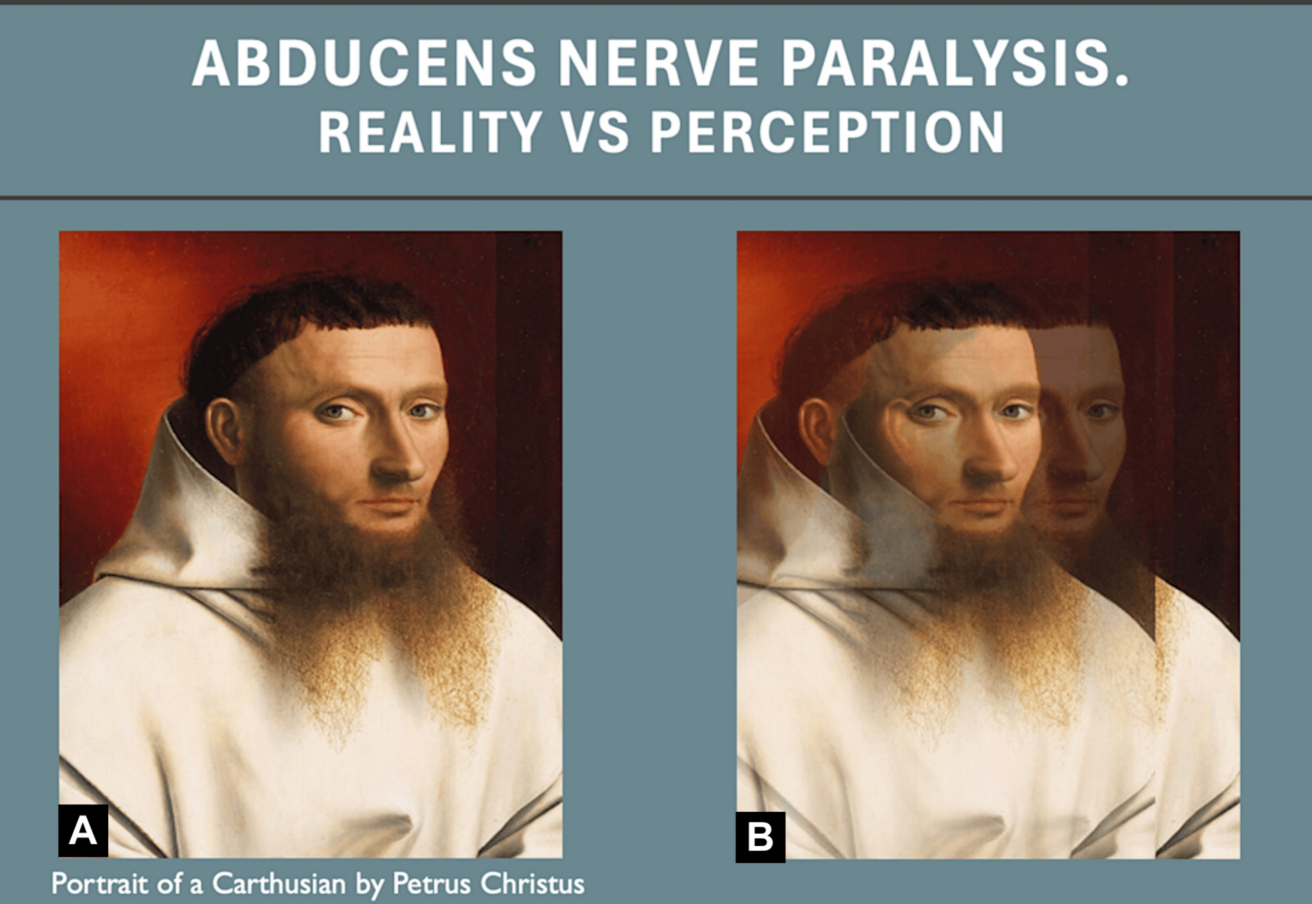
Reality vs. perception for a patient with abducens nerve paralysis. The image was generated by the students as part of the project. (A) "Portrait of Carthusian" by Petrus Christus [9], courtesy of The Metropolitan Museum of Art, New York, released under Creative Commons Zero (CC0). (B) The same image modified by the students with the help of Affinity Pro 2 to reflect the perception of a patient with abducens nerve paralysis.

The type of visual impairment experienced by this patient is presented in Figure 2.

Case 3. Cushing disease

Billy was diagnosed with a pituitary tumor five years ago. This non-cancerous tumor has led to an increased release of adrenocorticotropic hormone. This hormone, in turn, acts upon the adrenal gland to secrete the hormone cortisol, commonly referred to as the "stress hormone." Billy has had significant weight gain in the face and between the shoulders, as well as thinning skin that bruises easily. As a result of the increased cortisol levels, Billy has been diagnosed with central serous chorioretinopathy, a disease characterized by a buildup of fluid behind the retina.

This is what Billy says about his condition: "Throughout my day-to-day life, I find myself twisting and turning my head attempting to get a good view of the world through my peripheral. I was involved in various outdoor activities like bird watching and fishing; however, I can no longer see the tiny hummingbirds as easily because I cannot use binoculars anymore. The smallest activities, like putting an egg into a bowl to make scrambled eggs, have become difficult tasks."

**Figure 3:**
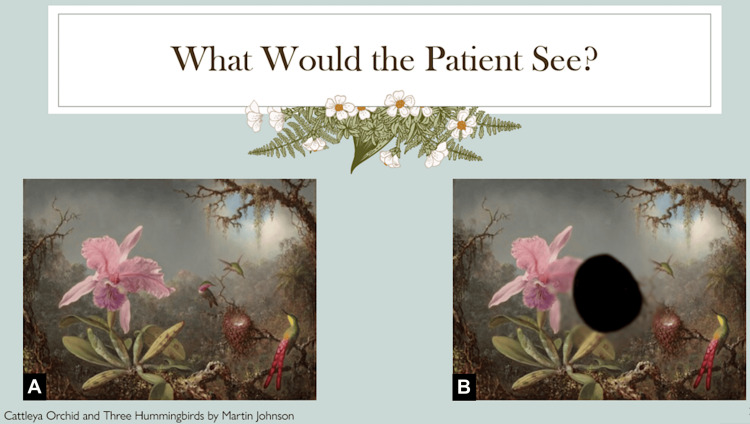
Reality vs. perception for a patient with Cushing disease. The image was generated by the students as part of the project. (A) "Cattleya Orchid and Three Hummingbirds" by Martin Johnson Heade [10], courtesy of the National Gallery of Art, Washington, released under Creative Commons Zero (CC0). (B) The same image modified by the students with the help of the Affinity Pro 2 software to reflect the perception of a patient with Cushing disease.

The type of visual impairment experienced by this patient is presented in Figure 3.

The post hoc assessment of the project's effectiveness and satisfaction was conducted. The response rate was 46%; 65% of participants agreed and strongly agreed that this assignment increased their knowledge and understanding of the disease mechanisms. Additionally, 77% of students agreed and strongly agreed that their perspective-taking skills had improved.

The common topics emerging from the narrative reflections were "clinical case presentation," "patient's perspective: understanding and explaining to others," "assumptions/stigmatization," "preparation for future profession," "pathological mechanisms," and "teamwork/collaborations."

Clinical case presentation

Students seemed to appreciate the opportunity to explore the underlying mechanisms of patients' visual abnormalities and the fact that pathological conditions not directly related to the visual system (i.e., cardiovascular and endocrine system pathology) can also cause vision impairment. This enhanced their understanding of physiology and added to the holistic approach to patient care. "I enjoyed the chance to dive deeply into the mechanism of … condition. This is not something we, as OD-1s, get to do very often. Mainly at this stage, we are just trained to recognize that a problem is present, so it was a good change of pace to get to learn the physiology behind a condition as well."

Patient's perspective

Students appreciated the opportunity to take the patient's perspective and acknowledged the fact that it was their first patient presentation. They liked seeing the world from the patient's perspective and understanding how their patients might feel. "I liked seeing it from the patient's perspective. Sometimes when you look only at the physiology/mechanisms behind, it is easy to overlook exactly how patients feel."

Assumptions/stigmatization

Students realized that their perceptions of how patients might feel and what they are able to do may be very different from reality. They understood that a lack of information often leads to patients' stigmatization and limits their access to certain jobs and roles in society. "I got to help my classmates understand that being color-blind doesn't have to limit what you do in life; it might even make you unique."

Preparation for future profession

Students mentioned the importance of this exercise in preparation for patient care and patient education. "I got to understand what color-blind people see, which can help me explain it to other people later on." Many students recognized the importance of teamwork for their future profession.

## Discussion

The present study suggests a novel approach to using visual art to teach empathy and explores the effect of this art-based exercise on self-reported empathy. The students reported high satisfaction with the project and a significant improvement in their perspective-taking skills. These results are supported by the positive effect of art-based projects on training cognitive empathy [2-6,11] and high levels of students' satisfaction with these interventions [11] reported by other researchers.

According to Zazulak et al. [12], cognitive aspects of empathy were enhanced even in the absence of an impact on the participants' overall empathic response.

In another study, Zazulak et al. [13] explored the impact of a course in arts-based visual literacy on the empathic response of medical residents engaged in obstetrics and gynecology and family medicine training. Although the majority of the psychometric measures did not reveal differences between groups throughout the program, thematic qualitative analysis of the interview data showed that the program had a positive impact on participants' perceived empathy towards colleagues and patients, as well as on their perception of personal and professional well-being.

Smith et al. [14] find the use of art-based training effective in addressing such emotionally complex medical problems as miscarriage.

Despite the overall positive evaluation of art-based interventions on empathy formation, the issues of consistency in such training, independent assessment of changed behaviors, and uncertain long-term effects persist [2]. At the same time, Lake et al. [15] state that the effectiveness of art interventions cannot be measured quantitatively and the possible outcomes of including arts in medical education are "an enriched view of lifelong learning and professional development, the potential to critique prevailing approaches to medical practice, and the revisualization of medicine as a succession of performances."

Limitations of the study

This study has certain limitations. It employed a quasi-experimental design without a control group, conducted in a single optometry school, which limits the generalizability of the results. Additionally, there was no standardized method for assessing empathy, and self-evaluation is susceptible to cognitive biases.

## Conclusions

Although the impact of this single intervention on student behavior was not studied and remains uncertain, it was well-received by students. They valued its relevance to their future careers, the group activity, and the chance to understand patient perspectives and medical conditions. We believe this assignment cultivates observation and interpretation skills, creates awareness of potential visual abnormalities in patients, fosters empathy and compassion, and aids in recognizing and mitigating biases. Given the importance and difficulty of teaching and assessing soft skills in healthcare professionals, early, systematic, and repeated training throughout the curriculum is crucial. Visual art, a powerful medium, should be increasingly utilized to teach cognitive empathy.
